# MicroRNA-196a promotes renal cancer cell migration and invasion by targeting BRAM1 to regulate SMAD and MAPK signaling pathways

**DOI:** 10.7150/ijbs.60805

**Published:** 2021-10-17

**Authors:** Jianzhou Cui, Yi Yuan, Muthu K. Shanmugam, Durkeshwari Anbalagan, Tuan Zea Tan, Gautam Sethi, Alan Prem Kumar, Lina H. K. Lim

**Affiliations:** 1 Department of Physiology , Yong Loo Lin School of Medicine, National University of Singapore, Singapore 117456, Singapore.; 2 NUS Immunology Programme, Life Sciences Institute, National University of Singapore, Singapore 117456, Singapore.; 3 Immunology Translational Research Program, Yong Loo Lin School of Medicine, National University of Singapore, Singapore 117597, Singapore.; 4 NUS Centre for Cancer Research, Yong Loo Lin School of Medicine, National University of Singapore, Singapore 117599, Singapore.; 5 Cancer Science Institute of Singapore, Yong Loo Lin School of Medicine, National University of Singapore, Singapore 117559, Singapore.; 6 Department of Pharmacology, Yong Loo Lin School of Medicine, National University of Singapore, Singapore 117559, Singapore.; 7 National University Cancer Institute, Singapore 119074, Singapore.

**Keywords:** MicroRNA-196a, renal cancer, Bram1, migration and invasion, SMAD and MAPK pathways

## Abstract

**Rationale:** MicroRNAs (miRNAs) are endogenous ~22nt RNAs that play critical regulatory roles in various biological and pathological processes, including various cancers. Their function in renal cancer has not been fully elucidated. It has been reported that miR-196a can act as oncogenes or as tumor suppressors depending on their target genes. However, the molecular target for miR-196a and the underlying mechanism in miR-196a promoted cell migration and invasion in renal cancer is still not clear.

**Methods:** The expression, survival and correlation between miR-196a and BRAM1 were investigated using TCGA analysis and validated by RT-PCR and western blot. To visualize the effect of Bram1 on tumor metastasis *in vivo*, NOD-SCID gamma (NSG) mice were intravenously injected with RCC4 cells (10^6^ cells/mouse) or RCC4 overexpressing Bram1. In addition, cell proliferation assays, migration and invasion assays were performed to examine the role of miR-196a in renal cells *in vitro*. Furthermore, immunoprecipitation was done to explore the binding targets of Bram1.

**Results:** TCGA gene expression data from renal clear cell carcinoma patients showed a lower level of Bram1 expression in patients' specimens compared to adjacent normal tissues. Moreover, Kaplan‑Meier survival data clearly show that high expression of Bram1correlates to poor prognosis in renal carcinoma patients. Our mouse metastasis model confirmed that Bram1 overexpression resulted in an inhibition in tumor metastasis. Target-prediction analysis and dual-luciferase reporter assay demonstrated that Bram1 is a direct target of miR-196a in renal cells. Further, our *in vitro* functional assays revealed that miR-196a promotes renal cell proliferation, migration, and invasion. Rescue of Bram1 expression reversed miR-196a-induced cell migration. MiR-196a promotes renal cancer cell migration by directly targeting Bram1 and inhibits Smad1/5/8 phosphorylation and MAPK pathways through BMPR1A and EGFR.

**Conclusions:** Our findings thus provide a new mechanism on the oncogenic role of miR-196a and the tumor-suppressive role of Bram1 in renal cancer cells. Dysregulated miR-196a and Bram1 represent potential prognostic biomarkers and may have therapeutic applications in renal cancer.

## Introduction

MicroRNAs (miRNAs) are endogenous ~22 nt RNAs that can play important regulatory roles in animals and plants. They can bind to their target mRNAs by complete or partial complementarily of their 5'-end nucleotides 2-8 (seed sequences) with a binding site in the 3'-UTRs of their target transcripts, which subsequently results in either direct cleavage of the targeted mRNAs by nucleases within the RISC or inhibition of their translation 1. Since their discovery in 1993 in *C. elegans*, these tiny oligonucleotides have been shown to play critical regulatory roles in a wide range of biological and pathological processes including cancer 2. Since 2002, an increasing number of miRNAs have been aberrantly expressed in various cancer cell lines and clinical tumor specimens. In general, miRNAs that are overexpressed in cancer are thought to act as oncogenic miRNAs by suppressing tumor suppressor, pro-apoptotic and growth-inhibitory genes. Down-regulated or absent miRNAs may present themselves as tumor suppressive as they usually repress oncogenes and proliferation-related genes 3. The identification of putative mRNA targets is important to understand the specific functions of miRNAs.

Renal cell carcinoma (RCC) represents the most common kidney malignancy and 3% of total human cancers. According to the WHO classification of renal cancer, there are five subtypes: clear cell, multilocular cystic, papillary, chromophobe, and collecting duct carcinomas 4. Research on miRNAs in renal cancer is less studied as compared to other cancers. The deregulation of miRNA has been found in all the subtypes of renal cancer cells 5, and the difference between specific miRNAs expression can be used to profile the five subtypes or as potential biomarkers in different stages of renal cancer carcinoma 6. In addition, by using the microarrays and RT-PCR, miRNA expression in a group of patients who developed metastasis compared with those who have non-metastatic tumors or primary RCCs was altered, such as miR-106b, miR-451, miR-221, miR-155 7-10. This suggests that the selected miRNAs might be potential biomarkers for metastasis of RCCs, but further studies and validations are needed.

MicroRNA-196a appears to play an important role in development and cancer. The up-regulation of miR-196a has been found in many cancers such as pancreatic cancer 11, breast cancer 12, esophageal adenocarcinoma 13, colorectal cancer 14, non-small cell lung carcinoma 15, gastric cancer 16 and intraductal papillary mucinous neoplasm 17. Although most studies on miR-196a suggest its oncogenic function in cancer, miR-196a may play a tumor suppressive role as well. For example, a strongly reduced expression of miR-196a was observed in melanoma cells compared to healthy control melanocytes 18, which may suggest that the functional role of miR-196a in cancer may be tissue- and cell type-specific. The mechanisms of action of miR-196a in different cancer types are largely unknown. It may depend on the molecules which miR-196a targets. It has been reported that miR-196a can directly target HOXA5 15, HOXA9 19, p27^kip1^ 20, ANXA1 21, ERG 22, NOBOX 23, FOXO1 24, Netrin 4 25, RAP1A 26, IκBα 27, radixin 28, and NFκBIA 29, or downregulation of HOXA7, HOXB8, HOXC8, HOXD8 14, HOXB7 18, CELF2 30 and NME4 31 and have several functions in different cancers. Also, miR-196a plays a functional role by regulating gene expression such as HOX-C8 32, HOX-B7, BMP4 18 and RAP1A 26. Liu et al. 15 demonstrated that miR-196a could promote cell invasion via targeting HOXA5, while Luthra et al. 21 suggested that the ectopic expression of miR-196a is associated with decreased cell migration. The effect of miR-196a is rescued by over-expressing ANXA1, which may also suggest that the functional role of miR-196a in cancer may be tissue- and cell type- specific. Thus, the function and targets of miR-196a in renal cancer cells remain unknown.

Bram1 (bone morphogenesis protein receptor associated molecule 1) is an alternatively spliced gene of BS69, which has been identified as a transcription suppressor gene 33. The C terminal of Bram1 is binds to BMP1A and may serve as an interacting protein in the BMP signal pathway 33, which is also confirmed in zebrafish 34. BRA-1, one homolog of Bram1 in *Caenorhabditis elegans*, binds to DAF-1 and negatively regulates transforming growth factor-beta (TGF-β) and DAF7 pathways 35. Besides, the MYND domain of Bram1 can also interact with the CTAR2 region of Epstein-Barr virus-encoded LMP1 (latent membrane protein 1), which acts as a constitutively activated tumor necrosis factor receptor (TNFR), therefore negatively regulates LMP1-mediated NFκB activation but not JNK activity 36. Bram1 was shown to be associated with the lymphotoxin beta receptor (LTβR), a TNFR-superfamily member. Bram1 reacts with LTβR mainly through the self-association domain of LTβR (aa 336-398), constitutively abrogates its function 37.

As mentioned above, miR-196a and Bram1 both showed an essential role in renal cancer cells. However, the correlation and underlying mechanisms between them remain unclear. Here, we performed a systematic, integrated analysis and investigated the role and target of miR-196a in renal cancer cells, namely BRAM1 and unveiled the mechanisms on how it plays a role as a tumor suppressor gene in renal cancer metastasis. Our results suggest that regulation of the renal miR-196a and Bram1 levels may be a novel therapeutic strategy for treating RCC.

## Materials and Methods

### Clinical database

The Bram1 gene expression in FPKM values of TCGA renal clear cell carcinoma (KIRC; N=534) available on Broad GDA, data version 2016_01_28 was extracted for analysis (https://gdac.broadinstitute.org/). The hsa-miR-196a expression in counts per million read were extracted from the KIPC and KIRP mi-RNA-seq.

### Cell culture

Human cell line HEK293, HEK293T, 786-O and Caki1 were obtained from American Type Culture Collection (ATCC, Manassas, VA, USA), RCC4 cell line was kindly provided by Dr. John Yuen, Department of Urology, Singapore General Hospital. Human embryonic cell line HEK293 (used for gene expression level examination), HEK293T (used for transfection experiments), human renal cancer cell lines RCC4 and 786-O were grown as monolayers in Dulbecco's Modified Eagle's Medium (DMEM, Serana, Australia) supplemented with 10% heat-inactivated fetal bovine serum (FBS, Biowest LLC, Kansas, MO, USA), 1% penicillin-streptomycin (GE Healthcare Life Sciences, HyClone Laboratories, Utah, USA). Human renal cancer cell line, Caki1 was cultured in RPMI (Serana, Australia) with 10% heat-inactivated FBS, 1% penicillin-streptomycin. All cell lines were kept at 37 °C in a humid atmosphere containing 5% CO2 (Thermo Fisher Scientific Inc, MA, USA).

### Cell transfection

The miR-196a sequences were amplified and cloned into pSilencer 4.1 CMV neo, and the wild type 3'UTR-Bram1 was cloned in to pSiCheck 2 vectors, pSiCheck-3'UTR-Bram1-mut were cloned by using the mutagenesis kit. According to the manufacturer's instruction, transfection (using Turbofect transfection reagent (Fermentas, USA) of the plasmids was performed in the various cell lines. pCMV-Bram1 and psupe-shBram1 and the negative controls are kind gifts from Dr. Yu-San Chang in Chang-Gung University. The sequences of all the plasmids were sent for sequencing in 1st Base company (Singapore) to ensure the constructs were correct. For plasmids transfection, empty GFP (500 ng) was co-transfected together with 1 µg of plasmids DNA in 12-well plates to assess transfection efficiency. After 24 hours, the cells were viewed using a fluorescent microscope at 10X magnification. At 36-48 hours after transfection, cells were harvested for Western blotting or RT-PCR analyses or other experiments.

For anti-miR transfection, human miRNA inhibitor negative control (5 nmol, mG/ZEN/mCmGmUmAmUmUmAmUmAmGmCmCmGmAmUmUmAmAmCmG/3ZEN/) and anti-hsa-196a (5 nmol, mC/ZEN/mCmCmAmAmCmAmAmCmAmUmGmAmAmAmCmUmAmCmCmU/3ZEN/) were synthesized in Integrated DNA Technologies (IDT, Singapore). RCC4 cells were transfected with hsa-196a anti-miR inhibitor (50 nM) and inhibitor negative control (50 nM) using 2 µL Lipofectamine™ RNAiMAX Transfection Reagent (Cat# 13778075) in 12-well plates.

### RNA extraction and RT-PCR

Total RNA was isolated with TRIzol reagent (Invitrogen, USA) according to the manufacture's protocol. miRNA was extracted using the NcleoSpin miRNA kit from Macherey-Nagel (Germany) according to the manufacture's protocol. Quantitative and qualitative RNA analyses were performed using NanoDrop 1000 spectrophotometer (Thermo Scientific). For samples to check gene levels, RNA was reverse transcribed to cDNA by using a Reverse Transctiption Kit (Promega). Real-time PCR (RT-PCR) analysis was conducted with GoTaq® qPCR master mix (Promega). For samples for microRNA study, cDNA synthesis and RT-PCR with the miRCURY LNATM system were conducted according to the manufacturer's protocol. Results were normalized to the expression of glyceraldehyde-3-phosphate dehydrogenase (GAPDH) or U6. RT-PCR and data collection were carried out on ABI7500.

### Western Blot assay

Cells were washed with cold PBS and lysed in mammalian protein extraction reagent RIPA buffer supplemented with protease and phosphatase inhibitors cocktail (Roche). Proteins were quantified by 1X Bradford's reagent (BioRad Protein Assay). Fifty microgram of total protein per samples were separated by 10% SDS polyacrylamide gel electrophoresis and transferred to a nitrocellulose membrane (BioRad). Proteins present in the membrane were incubated with specific antibodies and developed with a Konica Minolta SRX-101A tabletop processor. The antibodies used in our experiments included: Anti-Rabbit Bram1( Cell Signaling Technology (CST), Inc Danvers, MA, U.S.A); Anti-Rabbit BMPR1A (Gene tex Inc, San Antonio, Texas, U.S.A); Anti-Rabbit p-ERK (CST, Danvers, MA, U.S.A); Anti-Rabbit total-ERK (CST, Danvers, MA, U.S.A); Anti-Rabbit p-p38 (CST, Danvers, MA, U.S.A); Anti-Rabbit total-p38 (CST, Danvers, MA, U.S.A); Anti-Rabbit p-JNK (CST, Danvers, MA, U.S.A); Anti-Rabbit total-JNK (CST, Danvers, MA, U.S.A); Anti-Rabbit p-SMAD1/5 (CST, Danvers, MA, U.S.A); Anti-Rabbit EGFR (CST, Danvers, MA, U.S.A); Anti-Mouse Tubulin (Sigma-Aldrich Co St. Louis, MO, U.S.A.).

### Cell proliferation assays

5000 cells transfected with miR-196a or anti-miR-196a or negative controls cells were harvested and plated in 96-well plates containing normal media until visibly confluent (80%). Cell proliferation as well as cell counting assays were determined using CellTiter 96® (aqueous one solution cell proliferation) assay kit according to the manufacturer's instruction (Promega). Plates were incubated at 37 °C for 2 hr before reading OD at 490 nm.

For the colony-forming assay, 1000 cells transfected with miR-196a or anti-miR-196a or negative controls cells were harvested and plated in 6-well plates, after one week the cells were stained with 0.5% crystal violet (Amersco) and the pictures were captured using ChemiDoc™ Touch Imaging System (Bio-Rad). Cell Colonies were counted by using OpenCFU, an open-Source Software 38.

### Cell migration and invasion assay

Wound healing assay and transwell assay are used for detecting cell migration and invasion. For wound healing assay, 1×10^6^ cells were seeded in 6-well plates overnight and transfected with relative plasmids. Streaks were created in the monolayer with a pipette tip at 6 hr post transfection. Progression of wound closure was observed and photographed at 0 hr, 24 hr after wounding. For transwell assay, 24 hr after transfection with relative plasmids, 2.5×10^5^ cells in serum free medium were placed into the Matrigel transwell chambers consisting of polycarbonate membranes (8-µm pore size, millipore). Medium containing 10% FBS was added to the lower chambers. Following incubation for 48 hr, cells that remained on the top of the filter were removed and cells that migrated to the lower surface were counting.

### MiRNA target gene prediction

Correlation between miR-196a and gene expression data of predicted targets using Target Scan, miRWalk, DIANAmicroT and PicTar 39 were performed to find potential miR-196a target genes. The miR-196a mature sequences of human were obtained from the miRBase database.

### Dual luciferase reporter assay

Cells with 80% confluency were transfected with 100 ng of wide type or mutant plasmids in serum-free DMEM medium with and without miRNAs. After 24 hr, luciferase activity was determined using Dual- Luciferase® Reporter Assay System (Promega) according to the manufacturer's protocol. Cells were lysed in 120 μl of 1X lysis buffer, centrifuged at 10000 g for 10 minute and supernatants were collected. The results were expression as relative promoter luciferase activity compared to controls after normalizing for Renilla activity. Luminescence was measured using a spectrophotometer (Perkin Elmer VICTOR3™ V Multilabel Counter Model 1420).

### Immunoprecipitation

Cells were harvested in Immunoprecipitation (IP) lysis buffer and centrifuged at 4 °C. Concentration was quantified by BioRad Protein Assay and 500 μg of lysates were used for each reaction. Beads (20 μl) were washed and added to each lysate for pre-clearing, and 50 μl of beads were incubated with control IgG or specific antibody for 4 hour antibody-coupled beads. The pre-cleared lysates were mixed with antibody-coupled beads and incubated overnight. The next day, after washing the beads, 4X loading buffer was added to beads and heated at 95 °C for 10 min. Western Blot assay was followed to quantify the protein in the samples.

### *In vivo* metastasis model

All the procedures involving animals were reviewed and approved by National University of Singapore Institutional Animal Care and Use Committee (Protocol number R17-0975) and National Advisory Committee for Laboratory Animals Research (NACLAR). To test the effect of Bram1 on tumor metastasis, female NOD-SCID gamma (NSG) mice (*In vivo*s, Singapore) were injected with RCC4 cells (10^6^ cells/mouse) with stable empty vector or overexpression of Bram1 expression via tail vein (ten mice per group). After 4 weeks, mice were sacrificed; lung and liver metastatic nodules were detected in paraffin-embedded sections stained with hematoxylin and eosin following standard staining procedure. Images were taken using Leica DM6 microscope (magnification, 10×, 20× and 40×) and the images were captured using the Leica THUNDER imager.

### Statistical Analysis

Student's t-test (two-tailed) and One-way ANOVA were performed to analyze the data, p values less than 0.05 were considered statistically significant.

## Results

### Expression of BRAM1 in Kidney Renal Clear Cell Carcinoma (KIRC) TCGA cohort

**Figure 1:**
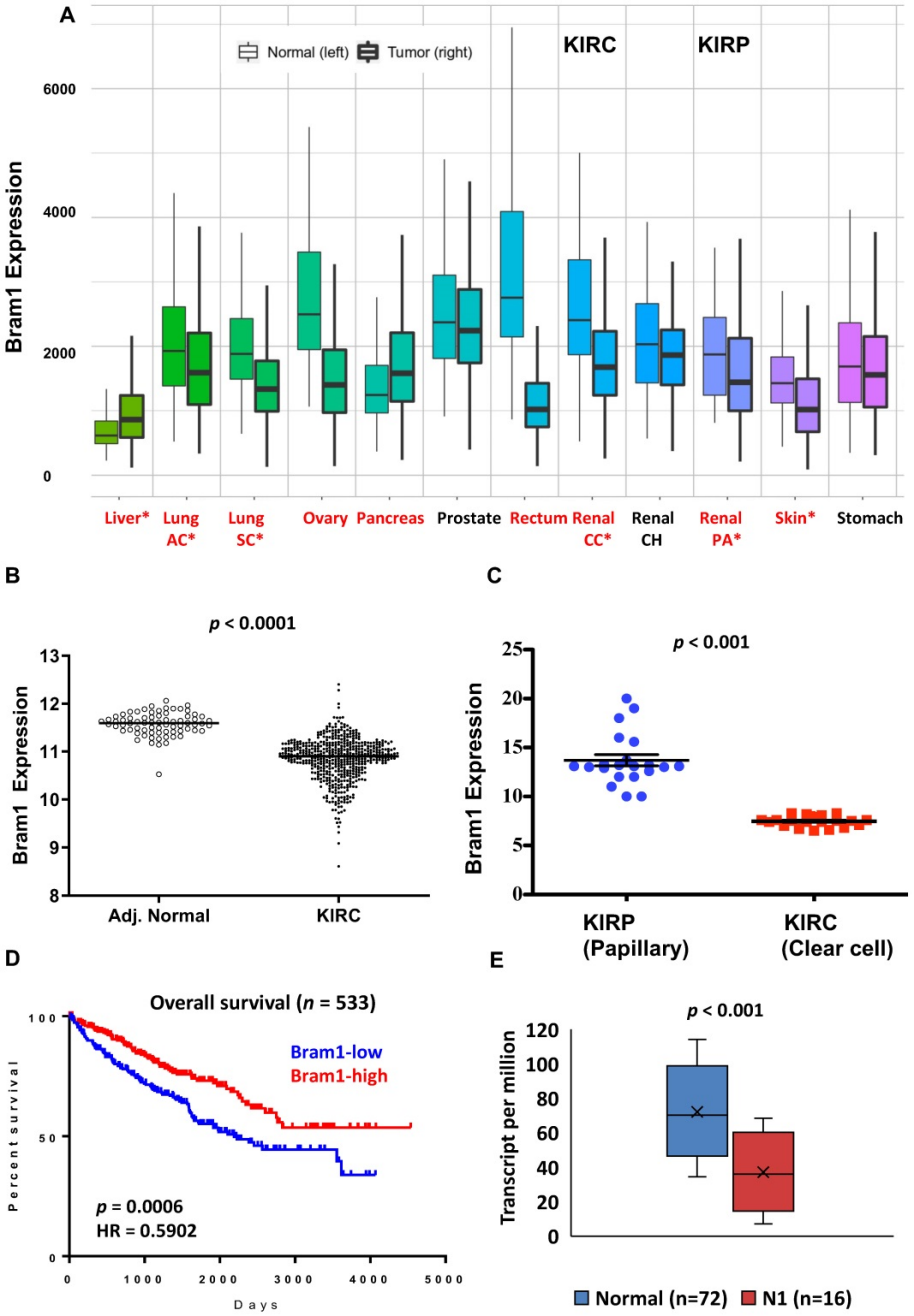
** Expression pattern of Bram1 in clinical samples. (A)** The gene expression pattern analysis of Bram1 in normal tissues and tumors based on TCGA data and **(B)** for KIRC.** (C)** Expression of Bram1 in clear cell renal cancer samples compared to papillary clinical samples. *p*<0.001. **(D)** Kaplan-Meier survival curves analysis for patients in TCGA renal clear cell carcinoma (KIRC) cohort (Red = high, blue = low; *p=*0.0006). **(E)** Expression of Bram1 in KIRC based on nodal metastasis status. Pathologic descriptions of N1: Metastases in 1 to 3 axillary lymph nodes.

We first determined the overall expression level changes in Bram1 (also known as ZMYND11) in all cancers using https://tnmplot.com/analysis/, which uses 56,938 unique multilevel quality controlled samples. **(Figure 1A)** 40. Similar to many other cancers, expression of BRAM1 was significantly reduced in renal clear cell carcinoma (light blue, CC) and papillary carcinoma (blue, PA). To confirm this, gene expression (FPKM) data from the TCGA renal clear cell carcinoma (KIRC) cohort was next downloaded from Broad GDAC Firehose (broadinstitute.org). The updated clinical outcome of TCGA cohorts were obtained from TCGA Pan-Cancer Clinical Data Resource. As shown in **Figure 1B and C**, lower Bram1 expression was detected in tumor samples and renal clear cell cancer samples compared with adjacent normal tissues and papillary clinical samples respectively. Kaplan‑Meier survival analyses demonstrated that the expression of Bram1 was positively associated with the survival of patients with KIRC **(Figure 1D)**. In addition, a lower level of Bram1 expression was detected in lymph node metastasis samples (N1, **Figure 1E**).

### Overexpression of Bram1 inhibits metastasis in mice

**Figure 2:**
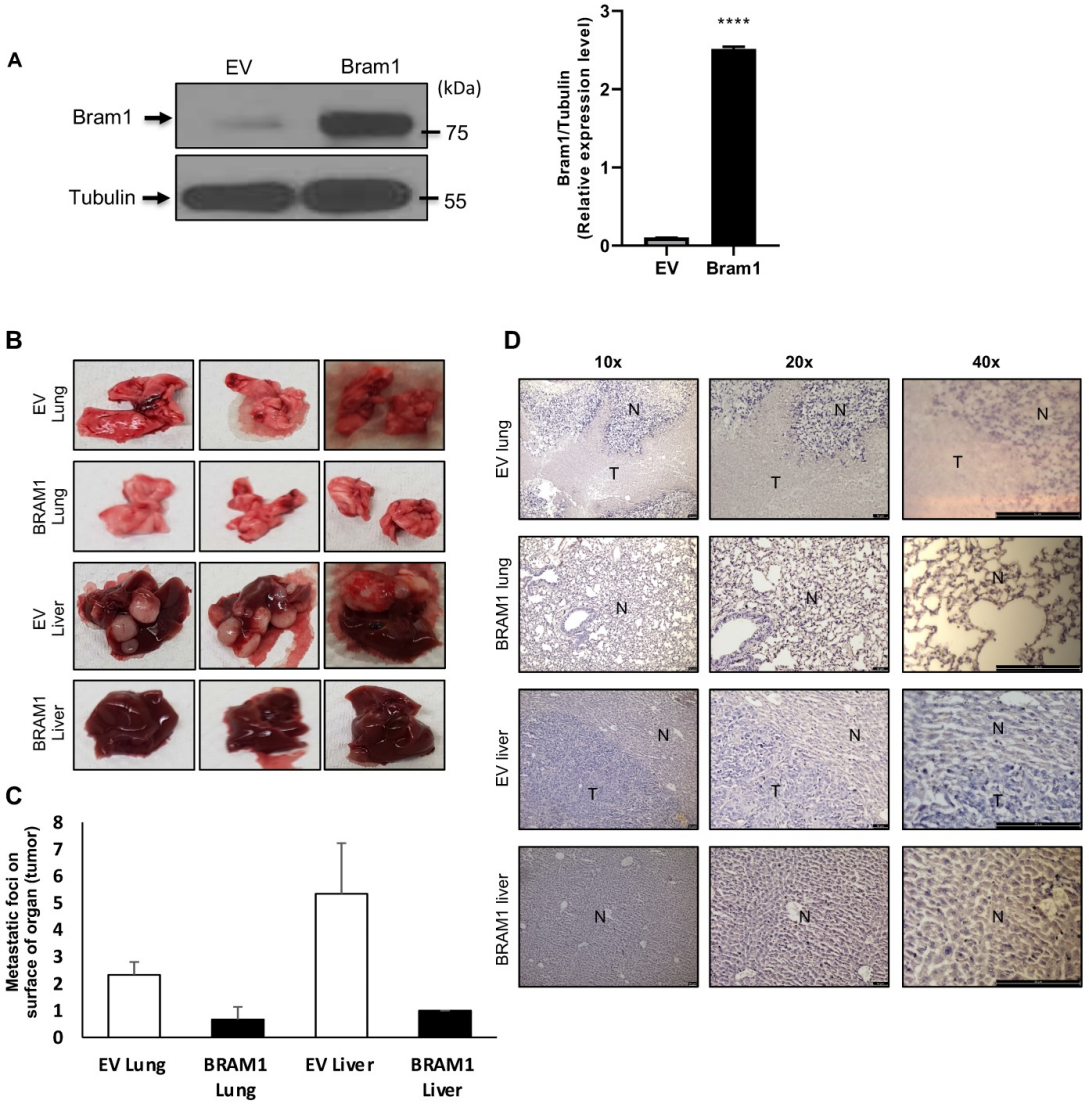
** Bram1 overexpression inhibits metastasis in mice. (A)** BRAM 1 expression in RCC4 cells stably transfected with empty vector or Bram1 overexpressing plasmids. **(B)** Representative images of metastatic lungs and livers of mice injected with Bram1 overexpression vector or empty vector (EV). **(C)** Quantification of the metastatic foci of the lung and liver of mice injected with Bram1 overexpression vector or empty vector (EV). **(D)** Representative images of hematoxylin and eosin staining of metastatic lung and liver samples collected from the control and Bram1 overexpressed groups (N=Normal, T=Tumor).

Next, we investigated whether overexpression of Bram1 expression could affect tumor metastasis in a xenograft metastasis model in which RCC4 cells were used to generate pulmonary and liver metastases. Stably transfected RCC4 cells were generated either with empty vector (EV) or Bram1 over-expression (OE). Overexpression level of Bram1 is confirmed via western blot as shown in **Figure 2A**. Strikingly, overexpression of Bram1 greatly suppressed lung and liver metastasis *in vivo*. The quantification of the metastatic foci indicated that Bram1-OE mice developed fewer metastatic foci on the lung and liver surface than EV mice **(Figure 2B, C)**. These observations were further confirmed by performing H&E staining **(Figure 2D),** which showed that the density of the lung and liver metastases in Bram1-OE cells was higher than that of the EV tissue samples. These data collectively suggested that high expression of Bram1 may prevent metastasis of renal cancer cells *in vivo*.

### The negative correlation between miR-196a and Bram1 in renal cancer cells

**Figure 3:**
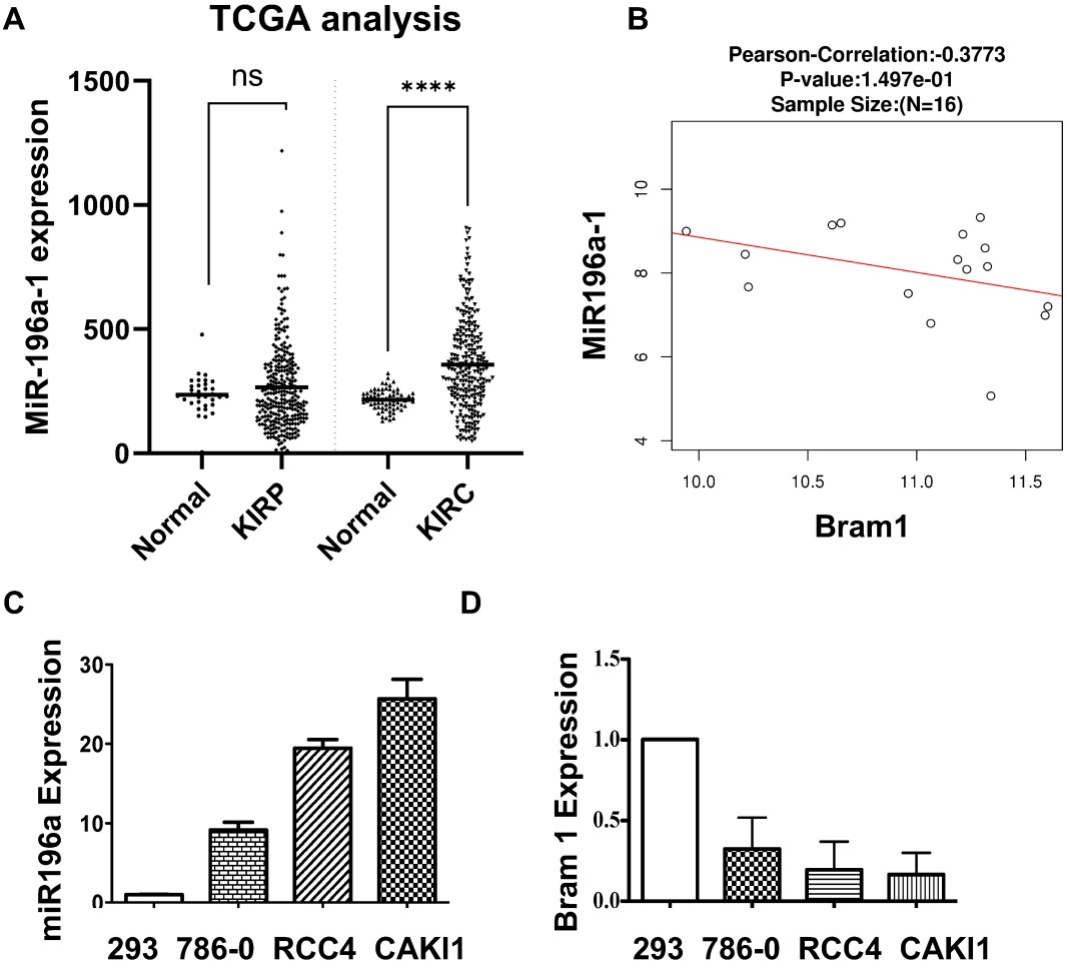
** Negative correlation between the miR-196a and Bram1. (A)** MiR-196a overexpression in clear cell renal cancer samples and papillary clinical samples. *p*= 1.69E-7. **(B)** The negative correlation between miR-196a and Bram1 expression in clinical samples. **(C, D)** The expression level of miR-196a and Bram1 was quantified by RT-PCR in renal cells.

MiR-196a has been shown to be down-regulated in metastatic renal cancer cells 9, yet the expression of miR-196a in primary renal cancer is not reported. Therefore, we investigated the expression of miR-196a in the database of renal cancer samples **(Figure 3A)**. Compared to the papillary group, miR-196a is significantly overexpressed in clear cell renal cancer cells**.** Further, a negative correlation between the miR-196a and Bram1 in renal tumors **(Figure 3B),** suggests that miR-196a and Bram1 play an inverse function in renal cancer cells. This can also be seen with BRAM1 expression in clinical samples, where Bram1 is expressed at lower levels **(Figure 1).** In cell lines, miR-196a was over-expressed in all the three renal cancer cell lines compared to HEK293 cells **(Figure 3C).** Based on the expression level of miR-196a in different renal cancer cells, miR-196a plasmids were overexpressed in HEK293T cells while anti-miR-196a were transfected in RCC4 cells for the subsequent experiments. We next determined the expression of Bram1 in RCC4, 786-O and Caki1 renal cancer cells. As expected, Bram1 was expressed lower in all the three renal cancer cells, compared to HEK293 cells **(Figure 3D),** while miR196a is expressed at a higher level. Thus, a negative correlation of miR196a and Bram1 expression, can be observed indicating that Bram1 might be a potential target for miR-196a.

### MiR-196a promotes embryonic renal cell proliferation, migration and invasion *in vitro*

**Figure 4:**
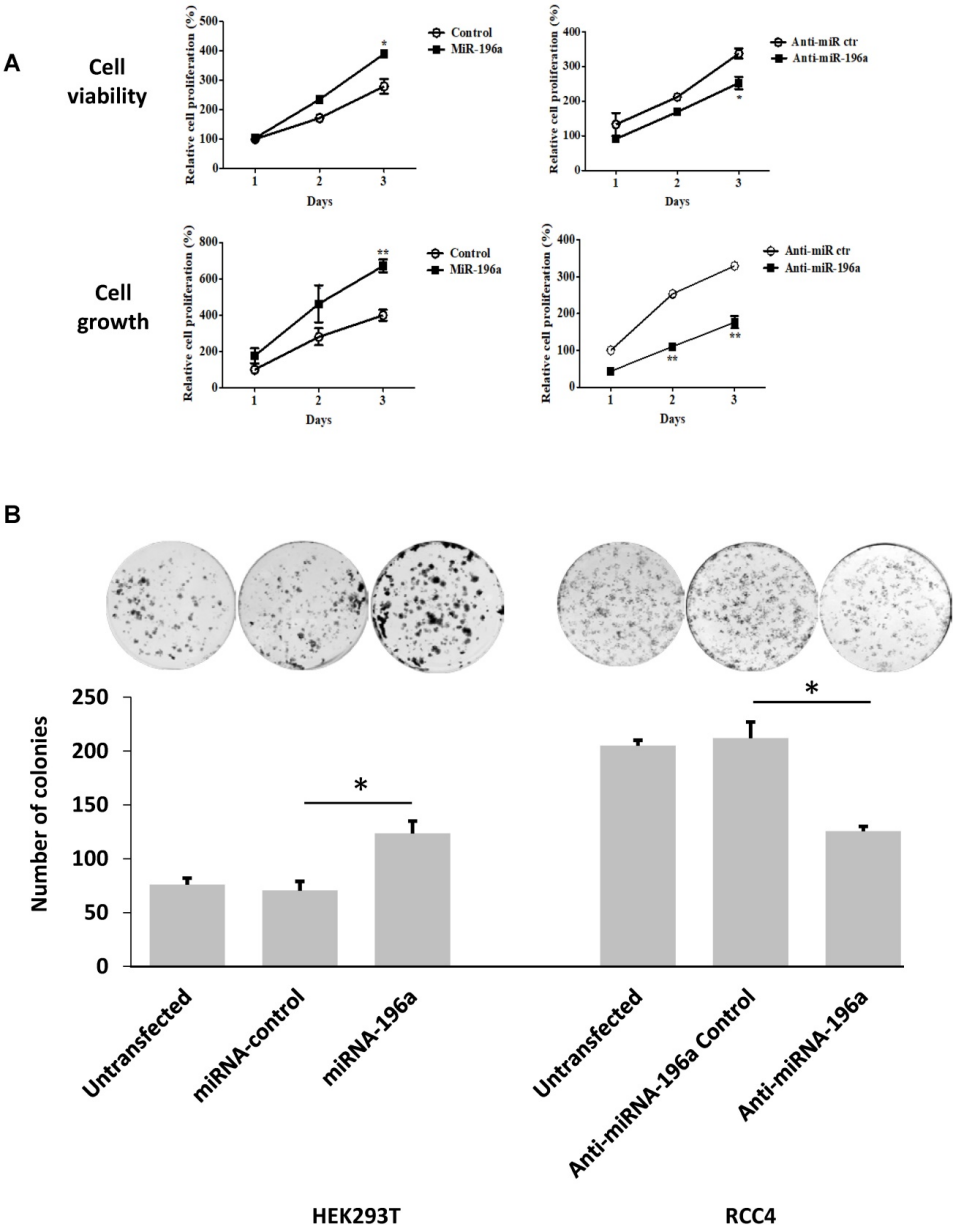

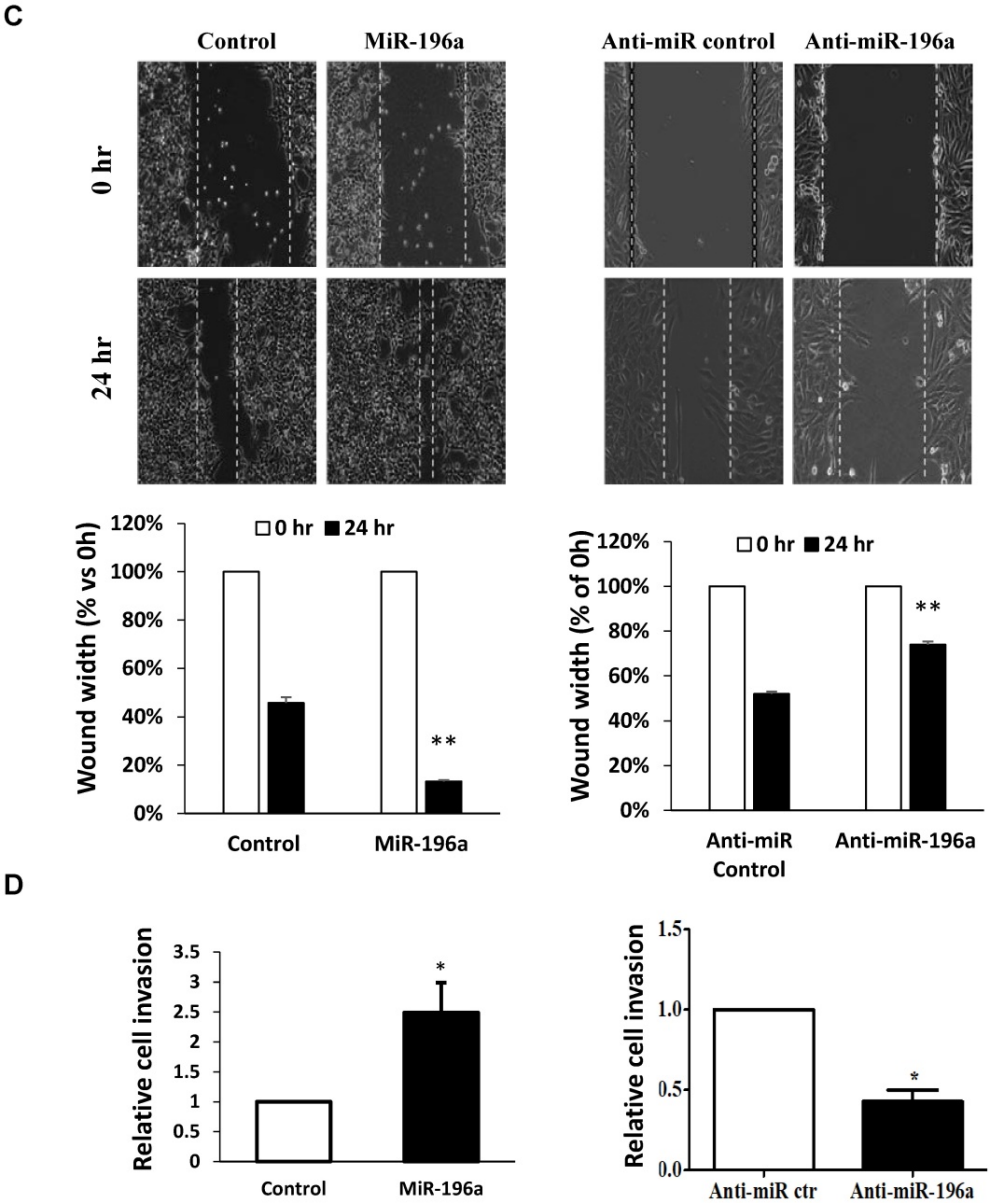
**MiR-196a plays an oncogenic role in renal cells *in vitro*. (A)** MiR-196a promotes cell proliferation in renal cells. HEK293T and RCC4 cells were transfected with miR-196a plasmids or anti-miR-196a nucleotides or negative control, followed by cell titer and crystal violet assay. Data is representative of three independent experiments. *P<0.05. **P<0.01. **(B)** The effect of miR-196a on colony forming ability in HEK293T and RCC4 cells. Untransfected cells and cells transfected with miR-196a plasmids or anti-miR-196a nucleotides or negative control were followed by crystal violet staining and imaging capture. Data is representative of three independent experiments. *P<0.05. **(C)** MiR-196a promotes renal cell migration by wound healing assay. HEK293T and RCC4 cells were transfected with miR-196a plasmids or anti-miR-196a nucleotides or negative control, followed by wound healing assay measured at 0 hr and 24 hr. The width of the wound in each well is quantified and presented as average ±SD. Data is representative for three independent experiments. **P<0.001. **(D)** HEK293T and RCC4 cells were transfected with miR-196a plasmids or anti-miR-196a nucleotides or negative control and seeded in the upper chamber of transwell plates. The cell number on the outside surface of this chamber was counted after 48 hours. Duplicates were performed in three independent experiments. *P<0.05.

Cell titer assay and cell counting assay were conducted to detect the effects of miR-196a on renal cell proliferation **(Figure 4A).** MiR-196a transfection into HEK293T significantly increased cell proliferation when compared to the control group in both assays. In contrast, RCC4 cells transiently transfected with anti-miR-196a nucleotides exhibited significantly lower cell proliferation compared to the cells transfected with anti-miR-control nucleotides. Furthermore, colony-formation capacity increased significantly after HEK293T cells were transfected with miR-196a. In contrast, when RCC4 cells transfected with anti-miR-196a nucleotides, the colony-formation capacity decreased significantly compared with untransfected cells and control cells **(Figure 4B).**

Next, the effect of miR-196a on renal cell migration and invasion was investigated via wound healing assay and trans-well assays. MiR-196a significantly enhances renal cell migration and invasion, while anti-miR-196a inhibits renal cancer cell migration **(Figure 4C)** and invasion **(Figure 4D).** Hence, we conclude that miR-196a promotes renal cell proliferation, migration, and invasion *in vitro.*

### Bram1 is a direct target of miR-196a in renal cells

**Figure 5:**
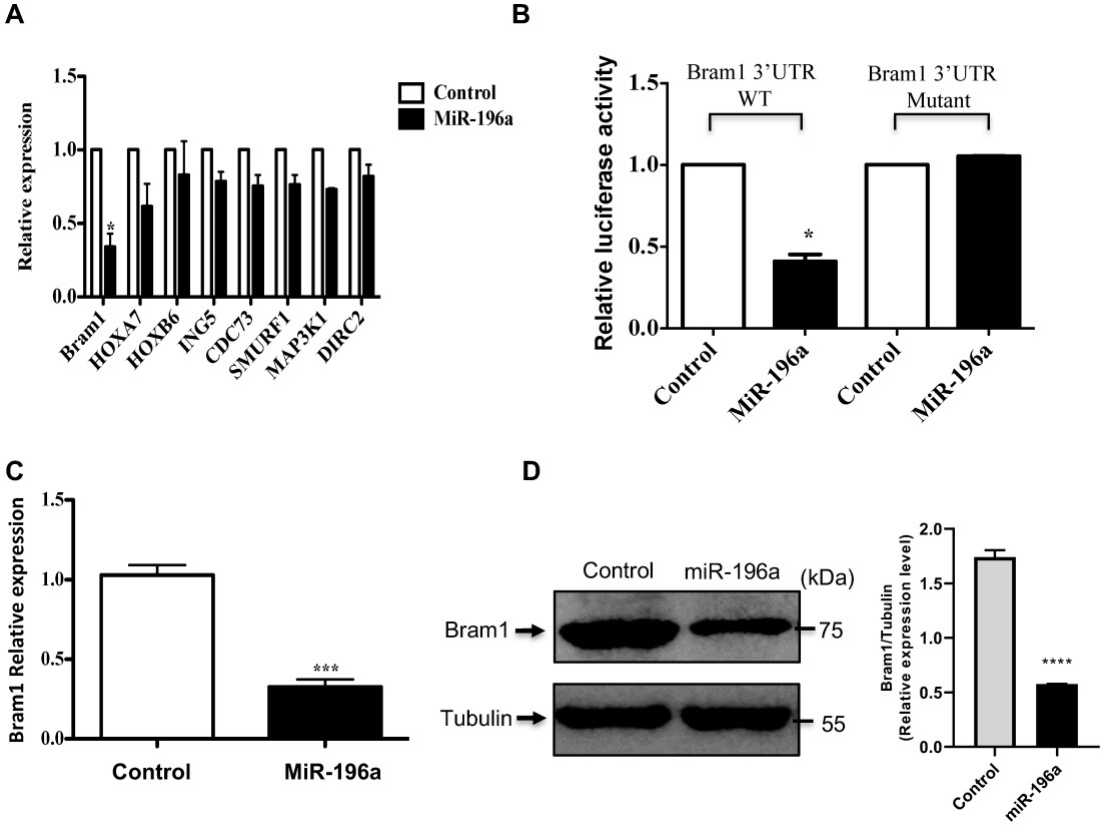
** Bram1 is a direct target of miR-196a. (A)** Gene screening for miR-196a candidate targets. HEK293T cells were transfected with miR-196a, and the expression level of indicated genes was quantified by RT-PCR. **(B)** The luciferase reporter containing wild-type Bram1 3'-UTR or mutant Bram1 3'-UTR was co-transfected into HEK293T cells with miR-196a plasmids and control. Luciferase activity was determined by the dual luciferase assay and normalized to Renilla activity. **(C, D)** HEK293T cells were transfected with empty vector or miR-196a, mRNA and protein expression were determined using RT-PCR and western blot. ***P<0.001.

Potential molecular targets for miR-196a were chosen using the microRNA target prediction software (Target Scan, miRWalk, DIANAmicroT and PicTar). Aside from those which have been reported, the top 8 candidates are listed according to their binding scores to miR-196a** (Table 1)**. Bram1 was shown to have the highest binding score to miR-196a among all these 8 candidates. To further investigate whether these targets are transcriptionally regulated by miR-196a, the expression of these candidate targets in miR-196a overexpressed HEK293T cells was quantified by RT-PCR **(Figure 5A).** Among all the 8 targets, only Bram1 was significantly inhibited with miR-196a transfection. This result confirmed the prediction analysis and suggests that Bram1 may be a candidate target for miR-196a in renal cancer cells.

Moreover, using the above target-predication softwares, the 3'UTR of Bram1 was scanned for predicted miR-196a target sites. The eight nucleotides comprising the seed sequences of miR-196a showed complete complementarity of two target sites in human 3'UTR of Bram1. To confirm that miR-196a directly targets Bram1, we cloned the wild-type and mutant 3'-UTR of Bram1 construct in psicheck2 vector **(Supplementary Figure 1).** Subsequently, these luciferase 3'-UTR constructs were co-transfected with miR-196a or empty vector plasmids into HEK293T cells. MiR-196a decreased the activity of the luciferase reporter containing the wild-type 3'-UTR of Bram1 mRNA, while no change for the luciferase reporter containing the mutant 3'-UTR of Bram1 mRNA **(Figure 5B).** We further confirmed that miR196a could regulate mRNA and protein expression of Bram1 using qPCR and western blotting in HEK293T cells transfected with miR-196a vectors. Indeed, Bram1 mRNA and protein was downregulated compared with cells transfected with control **(Figure 5C,D)**. This result confirms that miR-196a targets Bram1 by directly targeting its 3'-UTR.

### Bram1 inhibits renal cell migration and invasion, but not cell growth

**Figure 6:**
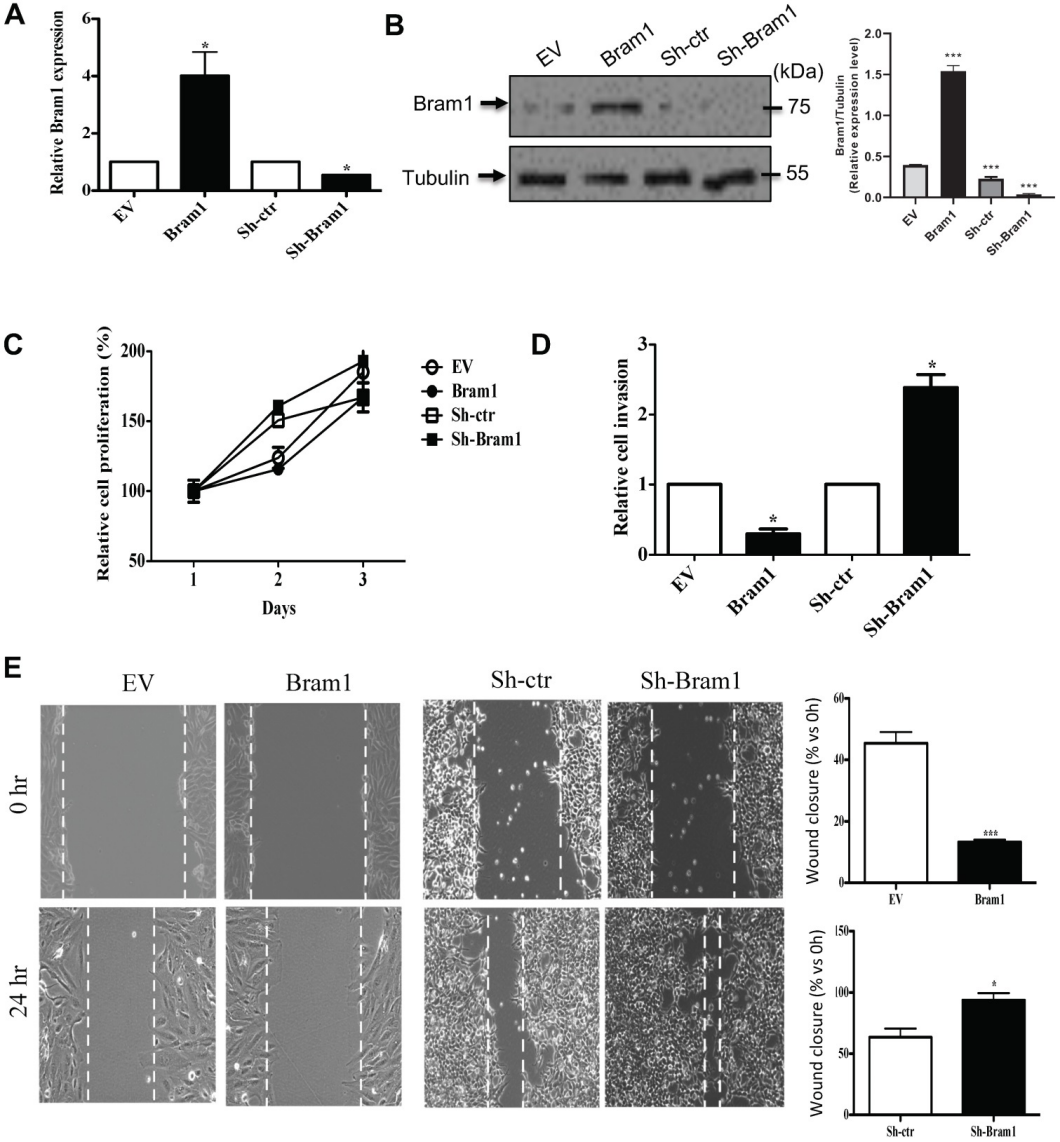
** Bram1 inhibits renal cell migration and invasion, but not cell growth. (A)** RCC4 and HEK293T cells were transfected with Bram1 plasmids or shBram1 plasmids and its relative control (EV or sh-ctr), mRNA level of Bram1 in these cells was detected by RT-PCR. Data is representative of three independent experiments. *P<0.05.** (B)** RCC4 and HEK293T cells were transfected with Bram1 plasmids or shBram1 plasmids and their relative control (EV or sh-ctr). The protein level of Bram1 in these cells was detected by western blot. **(C)** RCC4 and HEK293T cells were transfected with Bram1 plasmids or shBram1 plasmids and its relative control (EV or sh-ctr), followed by cell titer assay. **(D)** RCC4 and HEK293T cells were transfected with Bram1 plasmids or shBram1 plasmids and its relative control (EV or sh-ctr), followed by transwell assay. Duplicates were performed in three independent experiments. *P<0.05. **(E)** RCC4 and HEK293T cells were transfected with Bram1 plasmids or shBram1 plasmids and its relative control (EV or sh-ctr), followed by wound healing assay. The width of the wound in each well is quantified and presented in average ±SD. Data is representative of three independent experiments. *P<0.05. ***P<0.001.

We next determined the effect of Bram1 on renal cell proliferation and migration/invasion. HEK293T cells were transfected with either an EV or Bram1 or shBram1 constructs **(Figure 6A, B)** Cells transfected with Bram1 did not exhibit significant differences in cell proliferation compared with cells transfected with empty vectors indicating that Bram1 does not affect renal cell proliferation rates **(Figure 6C).** However, RCC4 cells transfected with Bram1 exhibited significantly less invasion than cells transfected with empty vectors **(Figure 6D).** Conversely, when HEK293T cells were silenced for Bram1, cell migration significantly increased. Similar results were obtained with a wound healing assay (**Figure 6E**). This data indicates that overexpression of Bram1 can inhibit migration and invasion of RCC4 cells, while silencing Bram1 increases HEK293T cell invasion and wound healing.

### Bram1 inhibits cell migration and invasion via MAPK and BMP pathways by binding to EGFR and BMPR1A

**Figure 7:**
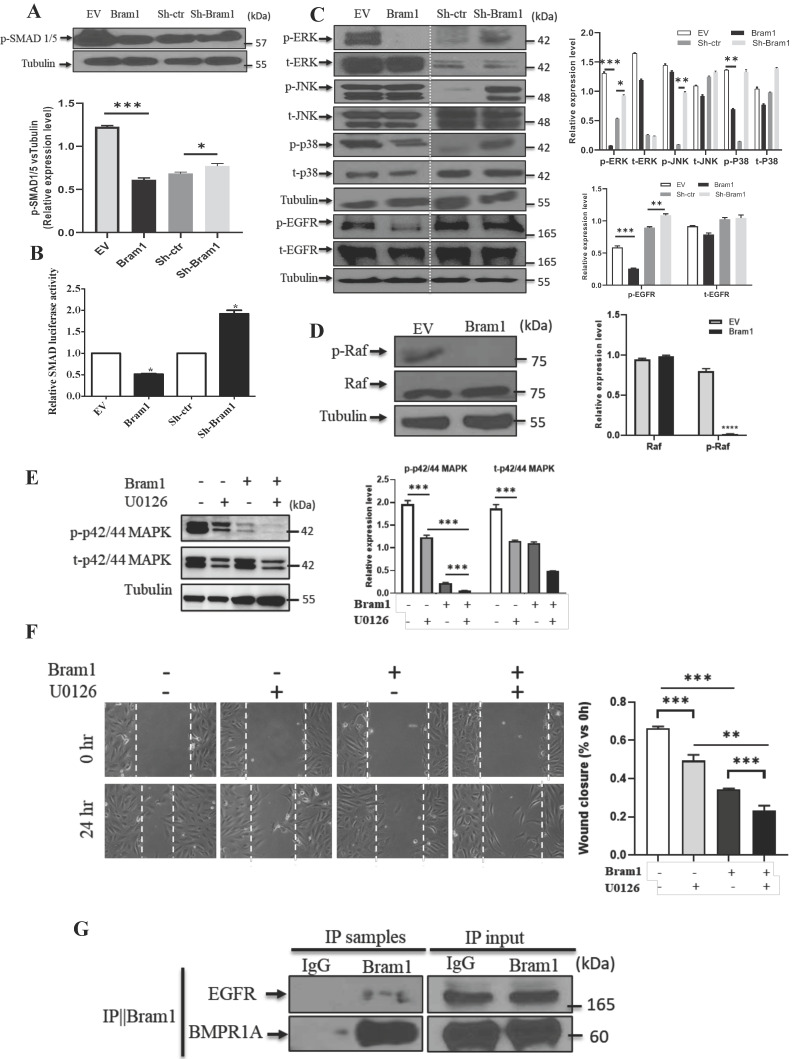

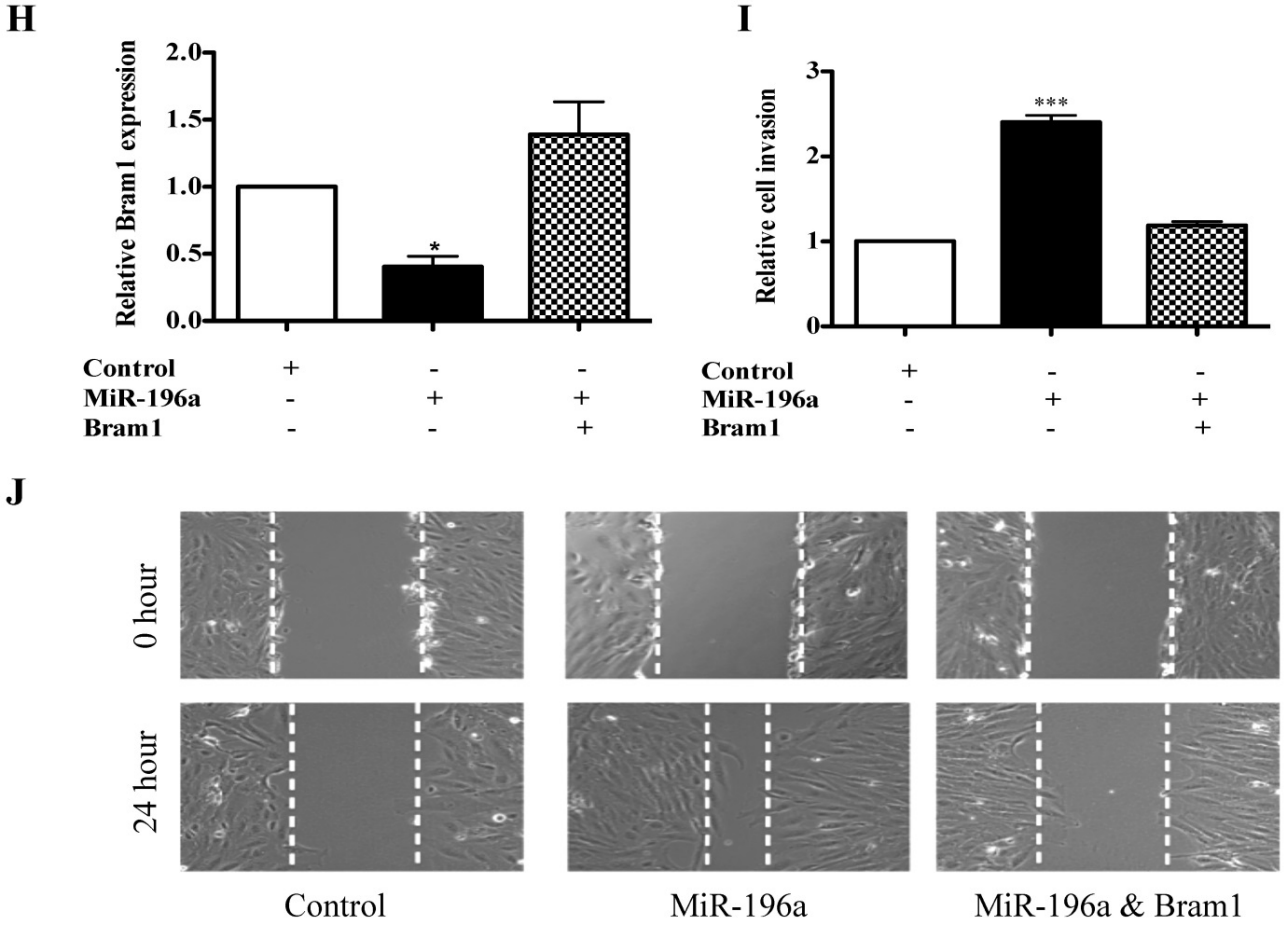
** Bram1 inhibits cell migration and invasion via MAPK and BMP pathways by binding to EGFR and BMPR1A. (A)** HEK293T cells were transfected with Bram1 or shBram1 and the expression level of phosphorylated SMAD1/5/8 and tubulin was examined by Western Blot assay. Data is representative of three independent experiments.** (B)** HEK293T cells were co-transfected with SMAD luciferase plasmids and Bram1 or shBram1 plasmids, and the lysate was used for luciferase assay. *P<0.05. **(C, D)** cells were transfected with Bram1 or shBram1, and the expression level of indicated proteins was examined by Western Blot assay. **(E)** Protein level of phosphate p42/44 and total p42/44 in empty vector (-) and Bram1 overexpression (+) RCC4 cells with or without U0126 treatment (10 µM, 4 hours). **(F)** Wound healing assay analysis the effect of MAPK inhibitor and Bram1 overexpression on cell migration (10 µM U0126 treatment for 4 hours).** (G)** The physical interaction between Bram1 and BMPR1A or EGFR was investigated by immunoprecipitation experiments. HEK293T cells were transfected with flag-Bram1, and treated with 50 µM BMP4, 48 hr post-transfection the lysates were collected and incubated with normal beads as a negative control or flag-beads. **(H)** RCC4 cells were co-transfected with miR-196a and Bram1 plasmids, the expression of Bram1 was detected by RT-PCR. *P<0.05. **(I)** RCC4 cells were co-transfected with miR-196a and Bram1 plasmids and seeded in the upper chamber of transwell plates. Duplicates were performed in three independent experiments. ***P<0.001. **(J)** RCC4 cells were co-transfected with miR-196a and Bram1 plasmids, followed by wound healing assay measured at 0 hr and 24 hr. Data is representative of three independent experiments.

Next, we investigated the mechanism of how Bram1 inhibits cell migration and invasion. Overexpression of Bram1 decreased the expression of p-SMAD1/5/8 in BMP pathway **(Figure 7A).** SMAD activity was quantified using the luciferase-expressing vector **(Figure 7B).** Overexpression of Bram1 in RCC4 cells significantly decreases SMAD luciferase activity, while silencing Bram1 in HEK293T cells results in higher SMAD luciferase activity, confirming that Bram1 can regulate the activation of SMAD. Besides, overexpression of Bram1 decreased the activation of ERK, p-EGFR and p38 MAPK **(Figure 7C),** while it did not affect p-JNK. Conversely, silencing Bram1 increases the expression level of p-ERK, p-JNK, p-EGFR and p-p38. Furthermore, overexpression of Bram1 decreased the expression of p-Raf **(Figure 7D).** To further investigate whether pharmacological inhibition of p42/p44 MAPK can affect the Bram1-induced cell migration suppression, the MEK inhibitor, U0126 was used **(Figure 7E, F).** UO126 further enhanced the Bram1 overexpression-induced cell migration suppression. These data indicate that Bram1 can inhibit cell migration via MAPK and BMP pathways.

Bram1 has been reported to bind to BMPR1A, which is the upstream protein of BMP pathway. Our immunoprecipitation data was shown that Bram1 could bind to BMPR1A. Moreover, Bram1 has been reported mainly to interact with membrane proteins, so we hypothesized that Bram1 can possibly interact with EGFR to inhibit the MAPK pathway. Immunoprecipitation experiments demonstrate that Bram1 physically interacts with both BMPR1A and EGFR **(Figure 7G).**

### MiR-196a-induced cell migration and invasion can be rescued by Bram1

To investigate whether Bram1 was involved in the increase in renal cancer cell migration induced by miR-196a, we performed co-transfection experiments. We found that co-transfection of Bram1 and miR-196a reversed the decreased expression of Bram1 protein induced by miR-196a **(Figure 7H).** Moreover, transwell assays **(Figure 7I)** and wound healing assays **(Figure 7J)** indicated that the co-transfection could partially rescue miR-196a-promoted migration in HEK293T cells. These data indicate that miR-196a could promote cell migration through the downregulation of Bram1 expression.

## Discussion

It has been reported that the expression of miR-196a in cancer is cancer-type specific 14, 15, 20, 31. Like many other miRNAs, miR196a is significantly reduced in metastatic vs primary renal tumors 9. The SNP (rs11614913) of miR-196a-2 is associated with RCC susceptibility and survival, serving as a biomarker for RCC occurrence and prognosis 5. Our results with renal cancer cells demonstrated that miR-196a is higher in all three cancer cells compared to normal human embryonic kidney HEK293T cells. This may be because all the cell lines used in our study (786-O, RCC4 and Caki1) are primary renal cancer cells and it is possible that miR-196a is increased in primary tumors and lost during the metastatic process of epithelial to mesenchymal transition (EMT). Although the underlying mechanism of the miRNA expression loss remains unclear, other studies have previously shown that the extracellular matrix crosslinking enzyme tissue transglutaminase-2 (TG2) only emerges in metastatic cells undergoing induction and reversion of epithelial-mesenchymal transition 41. In addition, spleen tyrosine kinase (SYK), TGF-β and P-bodies are also involved epithelial-mesenchymal plasticity and metastasis through autophagy in cancer cells 42, 43. Interestingly, epithelial to mesenchymal plasticity also enhanced fibronectin accumulation in paracrine and endocrine signaling, which regulates metastasis in breast cancer cells 44-46. This 'double face paradox' also exists in the expression of some other proteins, such as TGFβ. In the early stages of cancer, TGFβ exhibits tumor suppressive functions and inhibits epithelial cell cycle progression and promotes apoptosis. However, in the later stages of cancer, TGF-β1 is linked with increased tumor progression, higher cell motility, cancer invasiveness, and metastasis. It is also involved in the modification of the tumor microenvironment and promotion of EMT 47.

This hypothesis of overexpression of miR-196a during tumorigenesis and loss during metastasis can also be observed in breast cancer cells where MCF7 and T47D are seen to express higher levels of miR-196a compared to the more metastatic and invasive breast cancer cells MDA-MB-231, MDA-MB-435 and MDA-MB-468 cells. This seems to suggest that miR-196a is an oncogenic miR which can promote tumor growth, yet its loss in metastatic cells may indicate that it can also have negative roles in EMT, invasion or extravasation.

Molecular targets for miR-196a were discovered by microRNA target prediction software. Among these candidates, Bram1 was chosen for further study as it was the highest hit. Bram1 is a relatively novel gene, and this is the first study on Bram1 and miRNA regulation. A negative correlation was indeed observed in the expression level of Bram1 and miR-196a in renal cancer cells, and expression of miR-196a reduced Bram1 mRNA expression and protein levels. The 3'UTR luciferase assay showed miR-196a directly targets the 3'-UTR of Bram1, and Bram1 is involved in miR-196a-induced cell migration. Similar experiments have been done in other published studies, for example, Sox4, which is one target of miR-212, was shown to be involved in the miR-212 inhibitory tumor progression; p27^Kip1^, which is one target of miR-429, was observed to partially rescue the proliferation‑promoting effect of miR-429 in IA8 cells 48, 49.

Bram1 was shown to be highly expressed in renal cancer cells. Thus, the targeting of Bram1 by miR-196a might provide a mechanism on the modulation of Bram1 expression in renal cancer cells. Bram1, or otherwise known as ZMYND11, was identified in 1998 as an alternative spliced variant of BS69, which has been identified as a transcription suppressor gene 33. Bram1 mainly exists in the cytoplasm while BS69 exists in nucleus 50. Therefore, Bram1 may have different functions from BS69. Through its MYND domain in C-terminal, Bram1 has been reported to bind with some membrane proteins directly and regulate the relative pathway, such as BMPR1A 33, where it associates with BMPR1A in zebrafish and mammalian cells and blocks the BMP pathway. It can also inhibit NFκB activation by binding to LMP1 36 and LTβR 37. In *C. elegans*, BRA-1, a homolog of BRAM1, binds to DAF-1 and negatively regulates and DAF7 pathways, which are the homolog of TGF-β pathways 35. Bram1, as the target of miR-196 has been demonstrated in glioblastoma multiforme which revealed the important role of miR-196 and Bram1in cancer suppression 51 in cell growth and cell invasion. The underlying mechanisms and the downstream pathways remain unknown, however, although a previous study indicated that Bram1 suppressive effect on c-myb, myc and histone H3.3 might be considered 52-54.

In our study, Bram1 significantly inhibited cell migration in renal cancer cells, while it did not affect cell proliferation. To further investigate how Bram1 regulates migration, the main pathways involved in cancer migration were studied. Bram1 negatively regulated SMAD and ERK activation. The relationship between Bram1 and BMPR1A, which blocks SMAD pathway, was confirmed in this study by immunoprecipitation. This has previously been reported in different cells 33, 34. Moreover, the effect of inhibition of phosphorylation of various Smads on metastasis of RCC has been studied previously. Fibronectin silencing decreased cell growth and migration via suppression of the expression of cyclin D1, vimentin, and Smad3 phosphorylation in CAKI-1 and 786-O cells 55. Similarly, overexpression of CD82, also known as KAI-1, inhibited migration and invasion by suppressing phosphorylation of Smad2 and Smad3 and TGF-β1/Smad/MMP pathway in CAKI-1 cells.) These results are consistent with our finding in BRAM1, which suggest that CD82 and BRAM1 may inhibit RCC cells metastasis via inhibition of phosphorylation of various Smads 56.

As described above, cytosolic Bram1 has been shown to associate with membrane proteins. Therefore, we hypothesized that Bram1 might associate with the EGF receptor to inhibit MAPK activation as evidenced by immunoprecipitation assay, which has not been previously reported. Our studies confirmed that not only in GBM, but also for the other cancer cell lines, such as kidney cancers, the function of Bram1 as well as the role of miR-196 in cell progression, growth, migration, and invasion are functionally conserved. More importantly, we hereby demonstrate the potential underlying mechanism of miR-196 regulation of BRAM1 via SMAD and MAPK by directly binding to BMPR1A and EGFR, which contribute to the biological function mentioned above.

We have shown that the expression of miR-196a in primary clear cell renal cancer cells is up regulated, which suggests miR-196a might be a potential biomarker for primary renal cancer cells. Further, miR-196a plays an oncogenic role in renal cells, promoting cell proliferation and cell migration and invasion. Our study indicates that miR-196a may play a key role in RCC metastasis processes. The upregulated expression of miR-196a provides an important link to identifying suitable therapeutic targets (BRAM1) and diagnostic biomarkers for RCC. However, using a miRNA as a tumor marker often lacks specificity. The expression of miR-21 was significantly upregulated in RCC compared with healthy kidneys 58. However, other studies reported that RCC exhibited significantly lower expression levels of miR-21, -141 and -200b than normal tissues 59. These findings suggested that the combination of many miRNAs markers and other types of tumor markers and the targeting genes will significantly improve the accuracy of diagnosis 57. In terms of prognosis, more studies have showed that aberrant miRNA expression is associated with overall survival, disease grade and stage, recurrence, and metastasis 60. Since metastasis is common in RCC, miRNA196 expression and the negative correlation with BRAM1 expression indicates that miR-196a is associated with poor prognosis and may pave the way to clinical use of miRNAs as prognostic markers for metastasis. Due to the significant roles of miRNAs in the diagnosis and prognosis of cancer, increasing efforts are dedicated to the development of miRNA-based therapies. Either restoring functions of tumor-suppressive miRNAs or inhibiting oncogenic miRNAs could benefit cancer therapy. For example, silencing of miR-210 expression decreased the viability of ACHN and CAKI-2 cells and reduced the migratory and invasive potential of ACHN metastatic RCC cells 61. Hence, our results suggest that downregulation of miR-196 may suppress cell migration and act in a tumor-suppressive way in RCC. Moreover, miRNA-based therapies may also be used together with other therapeutic strategies, such as MAPK/ERK kinase inhibitors, in pre-clinical studies to enhance therapy strategies further.

## Conclusions

MiR-196a plays critical regulatory roles in renal cancer and the role of miR-196a in cancer is tissue- and cell type-specific. In the present work, miR-196a significantly induces renal cancer cell proliferation, migration and invasion by directly targets 3'UTR of Bram1 both *in vitro* and* in vivo.* Further investigation revealed that the underlining mechanism of Bram1 suppression is via the inhibition of Smad1/5/8 phosphorylation and MAPK pathway by directly binding to BMPR1A and EGFR. Thus, our results provide a new clue of the potential role of miR-196a in tumor metastasis and tumor suppression in renal cancer cells.

## Supplementary Material

Supplementary figure S1.

## Figures and Tables

**Table 1:** Candidate targets for miR-196a

Targeting gens	Target Scan	miRWalk	DIANAmicroT	PicTar
Bram1	100	0.923	1.000	6.56
HOXA7	100	0.846	1.000	3.25
HOXB6	91	-	0.991	3.22
ING5	78	0.864	0.881	2.17
SMURF1	55	0.471	-	-
CDC73	62	0.515	-	2.19
MAP3K1	84	0.500	0.981	2.80
DIRC2	51	-	-	-

4 microRNA target prediction software (Target Scan, miRWalk, DIANAmicroT and PicTar) were used for molecular target prediction for miR-196a. Besides the ones that have been reported, the others are listed in the table according to their binding score to microRNA.

## Abbreviations

BMP: Bone morphogenesis protein
BMPR1A: Bone morphogenesis protein receptor 1A
Bram1: Bone morphogenesis protein receptor associated molecule 1
EGF: Epidermal Growth Factor
EMT: Epithelial to mesenchymal transition
ERK: Extracellular signal-regulated kinases
GDAC: Global Disaster Alert and Coordination System
HOX: Homeobox miRNAs
miR: MicroRNAs
MAPK: Mitogen-activated protein kinases
MYND: Myeloid-Nervy-DEAF-1
RCC: Renal cell carcinoma
TGF- β: Transforming growth factor-beta
LTβR: Lymphotoxin beta receptor
UTR: Untranslated region
