# Supplementary material for: MicroRNA-196a promotes renal cancer cell migration and invasion by targeting BRAM1 to regulate SMAD and MAPK signaling pathways
Source: Int J Biol Sci. 2021 Oct 17;17(15):4254–70. doi: 10.7150/ijbs.60805 (PMC8579441; doi:10.7150/ijbs.60805)
Supplement: Supplementary file 1 — Supplementary figure S1. [file ijbsv17p4254s1.pdf]

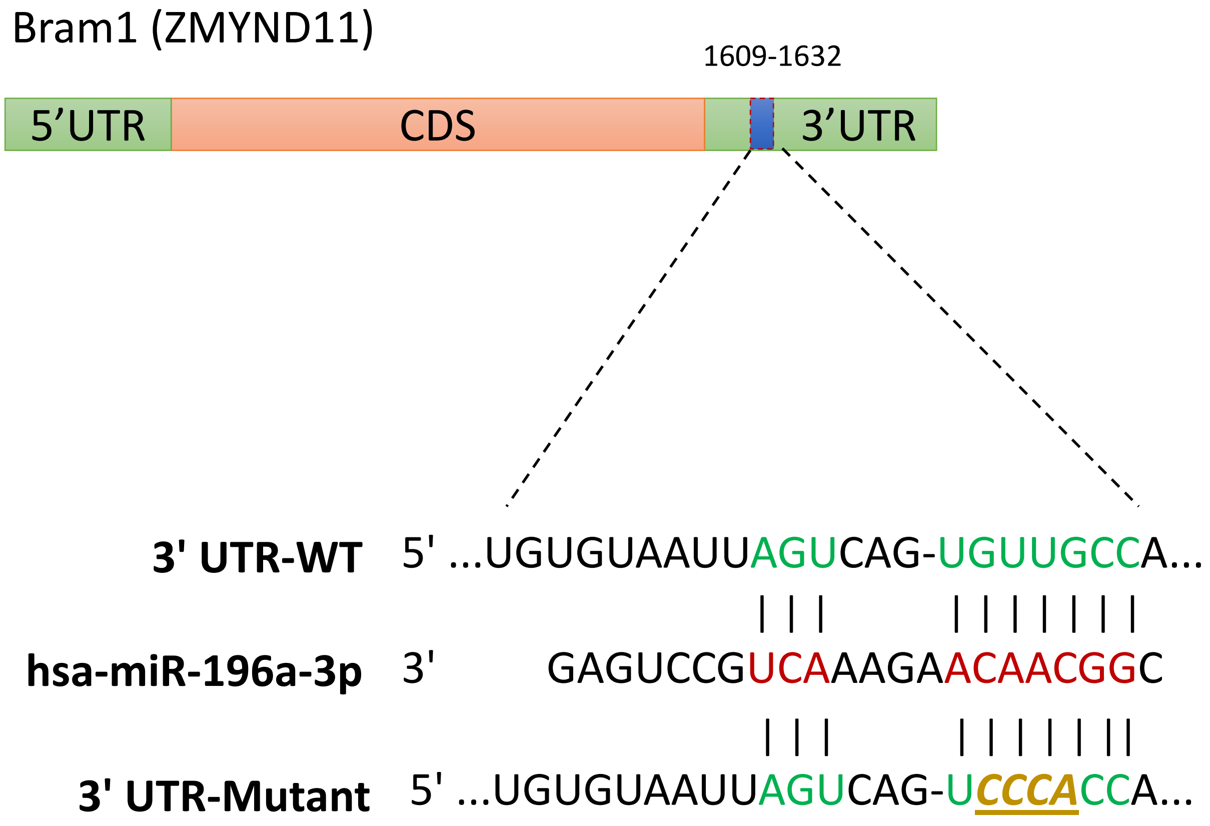

Supplementary Figure 1 Bram1 gene structure and miR-196a binding region Scheme shows pairing between miRNA-196a and Bram1 WT and mutated sequence, as predicted by TargetScan (http://www.targetscan.org/cgi-bin/targetscan/).
